# Rectangular plasmonic interferometer for high sensitive glycerol sensor

**DOI:** 10.1038/s41598-018-37499-2

**Published:** 2019-02-04

**Authors:** Zahra Khajemiri, Dukhyung Lee, Seyedeh Mehri Hamidi, Dai-Sik Kim

**Affiliations:** 1 Department of Physics, Shahid Beheshti University, G.C., Evin, Tehran, 19839 Iran; 2 Department of Physics and Astronomy, Seoul National University, Seoul, 151-747 Korea; 3 Magnetoplsamonic Lab, Laser and Plasma Research Institute, Shahid Beheshti University, Tehran, Iran

## Abstract

A novel plasmonic interferometric sensor intended for application to biochemical sensing has been investigated experimentally and theoretically. The sensor was included a slit surrounded by rectangular grooves using a thick gold film. A three-dimensional finite difference time-domain commercial software package was applied to simulate the structure. The Focused ion beam milling has been used as a mean to fabricate series of rectangular plasmonic interferometer with varying slit-groove distance L. Oscillation behavior is shown by transmission spectra in a broadband wavelength range between 400 nm and 800 nm in the distance between slit and grooves. Red-shifted interference spectrum is the result of increasing refractive indices. The proposed structure is functional from visible to near-infrared wavelength range and yields a sensitivity of 4923 nm/RIU and a figure of merit as high as 214 at 729 nm wavelength. In conclusion, this study indicates the possibility of fabricating a low cost, compact, and real-time high-throughput plasmonic interferometer.

## Introduction

The importance of glycerol detection as one of the most promising materials extends not only to experimental studies of flow characteristics^1^ such as biomicrofluidics and lab-on-a-chip applications, but also in other areas including food, pharmaceutical, leather, and textile industries^2^. Real-time detection and precise monitoring of glycerol is essential due to direct effect of glycerol concentrations in aqueous solutions on the quality of final products.

For this purpose, there are some techniques based on highly sensitive optical interrogation method where detection is performed using molecular binding interactions in surface binding modes such as Young interferometer^3^, Mach-Zehnder interferometer^4,5^, back-scattering interferometer^6^, Fabry–Perot Interferometer interferometer^7^.

As an ultra-sensitive, compact, real-time, and label-free biochemical sensor, surface plasmon polariton (SPP) based sensor is promising during recent years. SPPs can be recognized as a transverse-magnetic surface electromagnetic excitation propagating at the interface of a dielectric and a metal^8^. The interface of SPPs between metals consistently increases the sensitivity to change the refractive index of the dielectric material. Various methods have been introduced for SPPs excitation, such as prism-based Kretschmann configuration^9^, gratings^10,11^, and nanoplasmonic structures^12–16^. The origin of the surface plasmon resonance (SPR) relies on diffraction at prism or metallic gratings in a specific wavelength which results in coupling of the incident beams into propagating SPPs. The limitation of prism and grating coupling-based SPR techniques is associated to the production of SPPs only in a specific wavelength or a fixed angle of incidence^17^. The prism-based Kretschmann configuration is a popular SPR system which relies on light coupling on a flat metal film into SPPs, however, in spite of its high sensitivity, its bulky structure and high production cost decrease their practicality. To overcome these limitations, applying metallic nanostructures or nanoparticles in direct coupling of incident light into SPs has been developed in nanoplasmonic sensors^18–24^. Recently, the periodic-based nanoplasmonic structures have been used in biosensing applications^20–24^. However, the disadvantage of these structures is related to their low sensitivity compared to conventional SPR techniques^25,26^. A large resonance shifts in changing the refractive index can optimize the sensing performance of nanoplasmonic biosensors^27^.

Recently, plasmonic interferometers has become an area of increasing interest for refractive index sensing^4^, and medical imaging^28^. In an experimental Young’s double-slit-based study, the incorporation of a double-nanoslit structure upon the textured opaque gold film has been shown, in principle, to be efficient in propagating surface plasmon polaritons in the transmission of freestanding perforated gold film (Schouten *et al*.^29^). This followed the observation by Gao *et al*.^30^ that a self-referencing method including symmetric grooves near to the slit showed an improved interference pattern contrast owing to surface plasmon resonance sensing. The need for higher contrast and narrower linewidth have rekindled interest in development of apertures patterned on a gold-coated film in semicircular-based grooves forming a two-arm and three-beam plasmonic interferometer^31^. They showed that the design of their plasmonic interferometer allow a high flexibility in controlling the amplitude and phase of interfering SPPs as well as a narrow linewidth via the advantage of small size structure, however, the relatively low sensitivity (441.2 RIU/nm) limits its widespread application. Further, a recent work in the authors’ laboratory showed a high FOM in a small scale rectangular grooves-based plasmonic interferometer as compared with the aforementioned literatures, however, this structure also suffers from low sensitivity (500 nm/RIU)^32^. In this context, it is worthwhile to detect improved plasmonic interferometers that further enhance the sensitivity. In the present study, a collinear transmission configuration plasmonic interferometric sensor has been introduced theoretically and fabricate experimentally for higher sensitivity and real-time applications as compared with our earlier simulated work via the modification of rectangular grooves array around a slit.

## Results and Discussion

### Principle of rectangular plasmonic interferometer

The final intensity of this interferometer is given by^33^:

1 $$I={E}_{Light}^{2}+{E}_{spp}^{2}+2{E}_{Light}{E}_{spp}cos(\frac{4\pi L}{\lambda }{n}_{spp}+{\phi }_{0})$$

As a function of the incident electrical fields, E_Light_, SPP field, E_spp_, an efective refractive index of SPP, n_spp_ and an initial phase shift, φ_0_^32,34^. The proposed sensing scheme is based on spectral shift of the recorded pattern in broadband illuminations by changing refractive index. The main novelty of this plasmonic interferometer lies in effective refractive index detection and SPP excitation that allow maintaining real-time, and label-free sensing capability.

**Figure 1:**
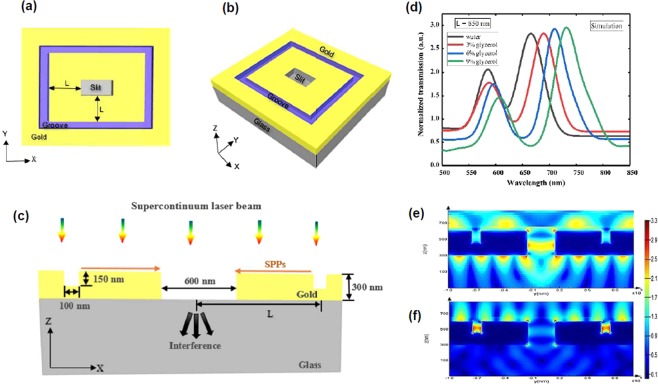
(**a**) Illustrates schematic of the proposed plasmonic interferometer, (**b**) shows profile xy (top view), (**c**) cross section view with wave propagation schematic in the center of the structure. (**d**) The simulated transmission spectrum of the proposed interferometer for different glycerol concentrations. The calculated electric field distributions at the interference peak (731 nm) and valley (665 nm) wavelengths for 9% glycerol–water for mixture L = 850 nm are plotted (**e**) and (**f**), respectively.

For simulation, the transmission spectrum of the structure was calculated with the three-dimensional finite difference time domain (FDTD) commercial software package (Lumerical Solution Inc.). The simulations are required firstly to determine the most appropriate groove depth and width to optimize the SPP coupling efficiency^35^. This simulation yielded an optimal geometric parameters set consisting of a slit with dimensions of 600, 300, and 300 nm in length, width, and depth, respectively, positioned in the middle of symmetric rectangular grooves with 100 nm and 150 nm width and depth respectively, at a distance L (Fig. 1a–c). By normal illumination of the broadband light source (400–800 nm), on the interferometer, the produced SPPs launched at grooves surface, and propagated along the slit. Therefore, the interference of transmitted SPPs and the incident light at the slit cause frequency dependent interference transmission (Fig. 1c). The simulation results shown in Fig. 1d–f illustrate the calculated distribution pattern of electric field at the interference peak and valley of 731 and 665 nm wavelengths respectively.

### Fabrication and characterization of rectangular plasmonic interferometer

**Figure 2:**
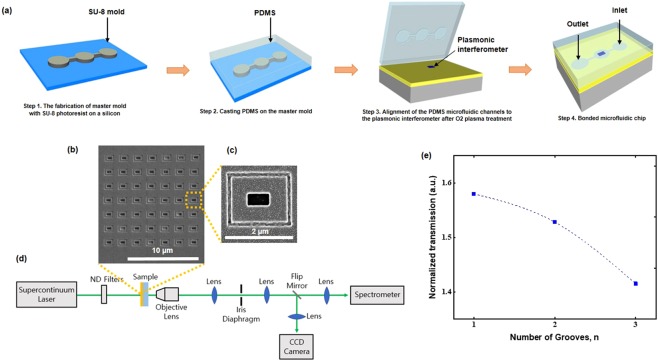
(**a**) Microfluidic fabrication process. (**b**) SEM image of the 7 × 7 fabricated plasmonic interferometer array. The center-to-center distance between each interferometer is 2 μm, and the sensor array footprint is 14 × 14 μm^2^. Scale bar: 10 μm. (**c**) One of the interferometers.Scale bar: 2 μm. (**d**) The shematic of our measurement. (**e**) Normalized transmission as a function of number of grooves, ‘n’ at period of 200 nm for L = 850 nm.

For experimental studies, the structures were milled in a 300 nm thick Au film deposited on a glass substrate using a focused ion beam (FIB). Details of the device fabrication are discussed in the Methods section. Figure 2b,c, show the SEM image of a rectangular plasmonic interferometer, consisting of a slit with dimensions of 600, 300, and 300 nm in length, width, and depth, respectively, positioned in the middle of symmetric rectangular grooves with 100 nm and 150 nm width and depth respectively, at the distance L = 850 nm. The designed interferometers have a 196 μm^2^ footprint with 2 μm distance between the centers of each sensing element. These dimensions give a 36 × 10^5^ potential packing density of sensors per cm^2^.

As shown in Fig. 2d, a supercontinuum laser beam (Fianium SC400-6) was normally incident on the sample from the slit side. Laser was unpolarized and it produced localized surface plasmons (LSPs) which was not correlated with TE polarization or TM polarization to produce LSPs. Here, SPPs were created from coupling between LSPs since SPPs could be produced when there was a coupling between LSPs forming chains^8^. The transmitted light was collected by a 40× microscope objective with the numerical aperture of 0.60. The collected light was coupled into a spectrometer (Ocean optics, USB2000) for spectral measurements. For identifying the positions of the rectangular plasmonic interferometers, a flip mirror was inserted to direct the sample image to another CCD camera (Sony XC-ES50).

In the present study the interactive effects between slit-grooves distances, refractive index, and groove numbers, and incident wavelength in a rectangular plasmonic interferometer were investigated. The calculation of groove periodicity (P) is described briefly as follows^36^:

2 $$P=\lambda {(\frac{{\varepsilon }_{m}+{n}^{2}}{{\varepsilon }_{m}{n}^{2}})}^{\frac{1}{2}}$$

Figure 2e shows the functionality of groove’s number. The peaks were located in the range of 710~730 nm. The increase in the number of grooves reduced the transmission spectra, based on resonant Bragg scattering, that generates SPPs for specific wavelengths^37^. As shown in Fig. 2e, the transmission through a one-groove structure could be much greater than predicted by two-groove or three-groove structures. Therefore, only one-groove was applied for all the samples.

### Refractive index sensing experiment

To illustrate the feasibility of our sensing scheme, the PDMS microfluidic (see microfluidic fabrication process in Fig. 2a and the details of fabrication discussed in the method section) flow was integrated on the device to inject the different ratios of glycerol-water concentrations. 0, 3, 6, and 9% glycerol–water mixtures with corresponding refractive index (RI) values of 1.331, 1.335, 1.340 and 1.344 were applied in the characterization.

**Figure 3:**
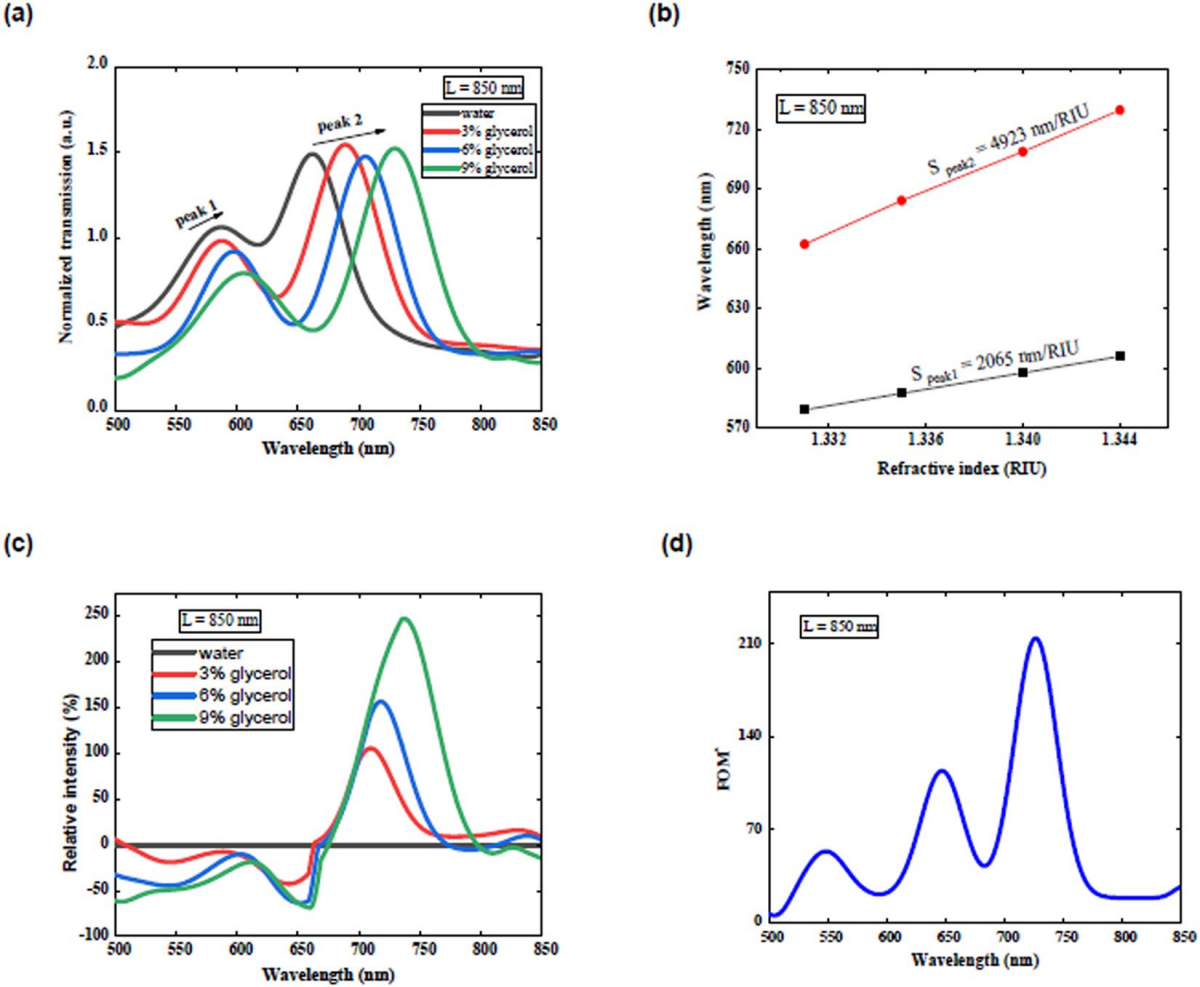
(**a**) Normalized transmission of a proposed structures measured at different refractive indices. (**b**) Peak wavelength as a function of the refractive index at the metal-dielectric interface. (**c**) The relative intensity changes for different refractive indices. (**d**) Calculated FOM* as a function of wavelength for rectangular interferometers for 3% glycerol–water mixture.

By normal illumination of the broadband light source on the interferometer, the produced SPPs traveled via the surface, and propagate to the slit, where SPPs and the direct light interfere together and modulate the far-field transmission. The SPP beam (E_SPP_) interfered with the incident beam (E_Light_) at the slit position. The calculated normalized intensity in L = 850 nm is presented in Fig. 3a. Red-shifted interference spectrum was the result of increasing refractive indices. Note that our proposed interferometric sensor operates over a broad spectral range, allowing to detect chemical analytes as a function of wavelength. This capability in the present device cannot be extended to other common SPR techniques based on prism and grating coupling approaches with the limitation of SPPs generation only at a specific wavelength^17^.

The sensitivity (S) of sensor can be defiened:

3 $${\rm{S}}=\frac{{\rm{\Delta }}\lambda }{{\rm{\Delta }}n}$$

As a function of spectral shift, Δλ, and the change of the surrounding refractive index, Δn^38^. The optical properties of our designed plasmonic sensor was explored based on different glycerol- water ratio. The peak positions around the wavelengths of 605 nm and 727 nm on the basis of the refractive index are presented in Fig. 3b. Employing the linear fits to the experimental data, the sensitivities for interferometer with L = 850 nm were obtained as 4923 (nm/RIU) and 2065 (nm/RIU) respectively at 729 nm and 605 nm which is in agreement with the observed simulation in the present study. These results are consistently greater than the reported values in the recent nanoplasmonic studies^9,11,13,19–25^ in this visible spectral region and other reported plasmonic interferometers^5,30–32,39–41^.

### The Figure of Merit of rectangular plasmonic interferometer

The relative intensity changes are shown in Fig. 3c. For liquids with different refractive indices, it given by:

4 $$\frac{{\rm{\Delta }}I}{{I}_{0}}=\frac{I-{I}_{0}}{{I}_{0}}$$

where I and I_0_ are the transmitted light intensity through the slit of a plasmonic interferometer at a specific glycerol concentration and the reference transmitted intensity of the sensor in water, respectively^37^.

To evaluate the sensing potential of the proposed structure with intensity interrogation, the figure of merit (FOM*) can be calculated as:

5 $${{\rm{FOM}}}^{\ast }=\mathop{\mathrm{lim}}\limits_{{\rm{\Delta }}n\to 0}(\frac{({\rm{\Delta }}I/{I}_{0})}{{\rm{\Delta }}n})$$

where ΔI/I_0_ and Δn show the change in relative intensity at an incident wavelength λ and change of the refractive index, respectively^38^. A high FOM* with a maximum value of 214 at wavelength λ of 727 nm is observed in Fig. 3d, which is in agreement with the observed simulation in the present study. This result is higher than the observed value in previous studies for nanoplasmonic^13,21,22^ in this visible spectral region and other types of plasmonic interferometer^30,31,39,41^.

**Figure 4:**
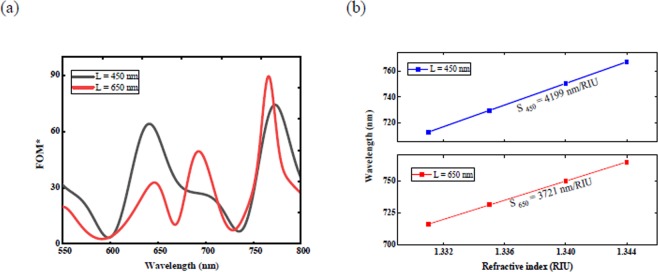
Calculated sensitivity and FOM* as a function of wavelength for rectangular interferometers for different L are plotted in (**a**) and (**b**), respectively.

The resulted FOM*s are 107 and 163, and the refractive index sensitivities are 4199 and 3721 nm/RIU, respectively for interferometer with L = 450 nm and 650 nm (Fig. 4a,b), which was in agreement with the results of the current study. The above observations are in accordance with the results of this study (see Table 1).

**Table 1:** Experimental and Calculated Sensor Sensitivities and FOM*s for rectangular plasmonic interferometer with Different L (Numbers in Italics are the simulation values).

L (nm)	Wavelength (nm)	Sensitivity (nm/RIU)	FOM*
450	767	*4199*	*107*
		4007	101
650	764	3721	163
		*4121*	*158*
850	729	4923	214
		*4615*	*230*

### Real-time Sensing

**Figure 5:**
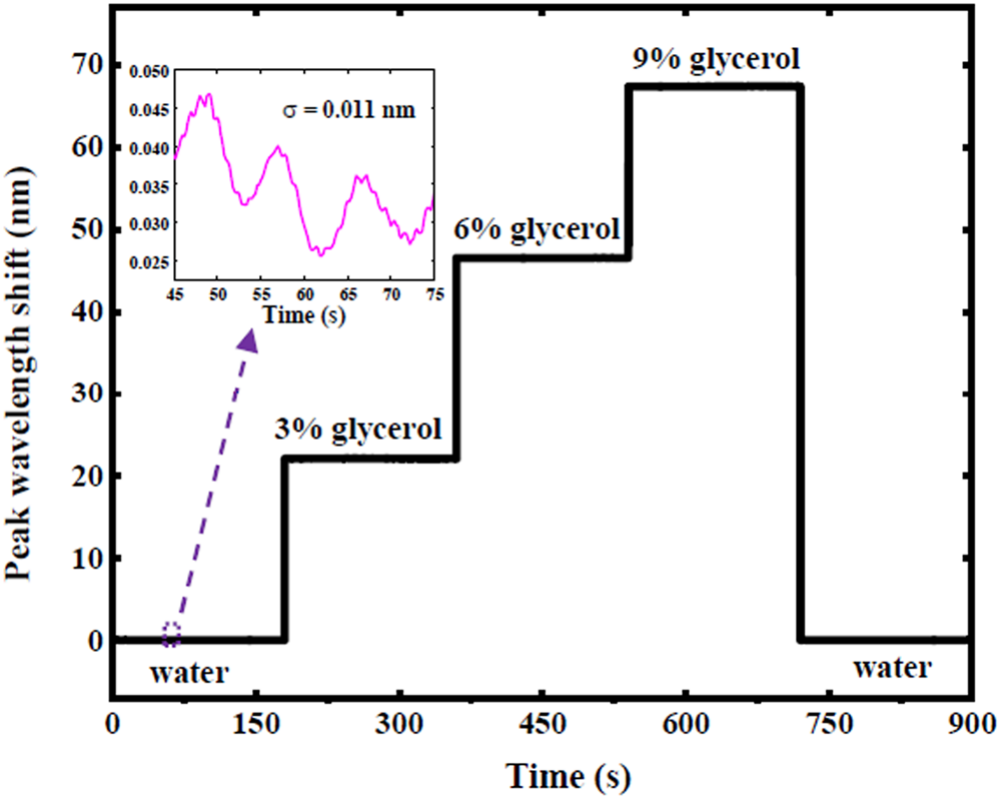
The real-time measurements of peak wavelength shift from interferometer.

A real-time experimental measurement is shown in Fig. 5, in which the value of transmitted intensities was measured as L= 850 nm. The PDMS microfluidic flow was integrated on the device to inject the different ratios of glycerol-water concentrations with an interference peak around 729 nm. Afterward, Lorentzian fitting method^26^ was used to evaluate and plot the peak position as a function of time (Fig. 5). For real-time refractive index sensing trial, the flow of temporal resolution over the sensor was 10 s for the continuously recorded transmission spectra. A stable peak wavelengths exhibited at each glycerol concentration and the increase in glycerol concentrations proportionally increased the peak shifts. The final DI water injection decreased the peaks to their initial spectral positions. The standard deviation of peak wavelength shift was calculated and shown in the upper inset of Fig. 5. A sensor resolution was 2.2 × 10^−6^ RIU (0.011 nm/4923 nm/RIU). This result is higher than the observed value in previous studies for nanoplasmonic^13^, nanohole array sensors^42^ and other plasmonic interferometer^5,30^. However, further resolution improvement could be obtained by using ultrasmooth metal films to decrease the SPP propagation loss and enhance the sensitivity^43^.

## Conclusion

In conclusion, novel plasmonic interferometers have been proposed based on optimized incident beam and interference of SPP, which can cover the entire visible and near‐infrared Our study indicates a high sensitivity of interference peaks to geometrical structure, particularly in detection of refractive indices in dielectric materials. A sensitivity of 4923 nm/RIU with a resolution of 2.2 × 10^−6^ RIU in 14 × 14 μm^2^ sensing area has been experimentally demonstrated. This may provide a new candidate for real-time, small size, and low cost bio-sensing device.

## Methods

### Fabrication of plasmonic interferometers

A 5nm-thin chrome was deposited by e-beam evaporation (KVE-E4000, Korea vaccum tech) on a glass substrate as an adhesion layer and subsequently a 300-nm-thick gold film was placed directly onto glass slides at 0.5 Å/s deposition rate. Before the evaporation, acetone and isopropyl alcohol and deionized water were used as detergents to clean the glass slides with an ultrasonic cleaner successively for 10 min, and then dried with nitrogen. Focused ion beam (Helios 650; FEI, USA) milling (30 kV, 30 pA) was used to fabricate a series of rectangular plasmonic interferometer with varying slit-groove distance L.

### Fabrication of PDMS Microfluidic Channels

Conventional soft lithography was applied to fabricate microfluidic channels (Fig. 2a). A SU-8 (2010, Microchem) mold with a 50 µm depth and 100 µm width was patterned on a 4-inch silicon to form microfluidic channels (Fig. 2a, step 1). A 10:1 mixture ratio of PDMS (Sylgard 184, Dow corning) and curing agent was applied to cast the mold (Fig. 2a, Step 2). The mixture was baked at 120 °C for 30 minutes. After cooling, the PDMS channel was cut and peeled, afterwards inlet and outlet holes were punched. Next, the PDMS channel and the nanopatterned sample were activated by oxygen plasma treatment (15KVS034, Korea vaccum tech., LTD; Fig. 2a, step 3) and finally bonded together (Fig. 2a, step 4).

### Numerical method

The transmission spectrum of the structure was calculated with the three-dimensional finite difference time domain (FDTD) commercial software package (Lumerical Solution Inc.). The incidence of plane wave was normally began from the top and polarized along the x-axis, as shown in the Fig. 1c. The periodic boundary conditions were detected for x axis and y axis, and perfectly matched layer (PML) was observed for the boundary conditions of z axis. A mesh size of 10 nm was utilized. In order to monitor the electric field profile and transmission spectra, a detector was situated at suitable position.
